# Efficacy and safety of isatuximab plus bortezomib, lenalidomide, and dexamethasone in patients with newly diagnosed multiple myeloma ineligible/with no immediate intent for autologous stem cell transplantation

**DOI:** 10.1038/s41375-023-01936-7

**Published:** 2023-06-14

**Authors:** Enrique M. Ocio, Aurore Perrot, Pierre Bories, Jesus F. San-Miguel, Igor W. Blau, Lionel Karlin, Joaquin Martinez-Lopez, Song-Yau Wang, Sara Bringhen, Magda Marcatti, María-Victoria Mateos, Paula Rodriguez-Otero, Stefania Oliva, Axel Nogai, Nadia Le Roux, Liyan Dong, Sandrine Macé, Matthieu Gassiot, Thomas Fitzmaurice, Corina Oprea, Philippe Moreau

**Affiliations:** 1 University Hospital Marqués de Valdecilla (IDIVAL), University of Cantabria, Santander, Spain; 2 CHU de Toulouse, IUCT-O, Université de Toulouse, UPS, Service d’Hématologie, Toulouse, France; 3 Toulouse University Institute of Cancer-Oncopole, Toulouse, France; 4 Clinica Universidad de Navarra, CCUN, CIMA, IDISNA, CIBERONC, Pamplona, Spain; 5 Charité Medical University, Berlin, Germany; 6 Hôpital Lyon Sud, Hospices Civils de Lyon, Pierre-Bénite, France; 7 Hospital 12 de Octubre, Complutense University, CNIO, Madrid, Spain; 8 Department of Hematology and Oncology, University of Leipzig, Leipzig, Germany; 9 SSD Clinical Trial in Oncoematologia e Mieloma Multiplo, Division of Hematology, University of Torino, Azienda Ospedaliero-Universitaria Città della Salute e della Scienza di Torino, Torino, Italy; 10 Vita-Salute San Raffaele University, Milan, Italy; 11 University Hospital of Salamanca, IBSAL, CSIC/CIC, Salamanca, Spain; 12 Dipartimento di Oncologia ed Ematologia SC Ematologia 1 U, Torino, Italy; 13 Sanofi Research & Development on behalf of Altran, Vitry-sur-Seine, France; 14 Sanofi, Beijing, China; 15 Sanofi Translational Medicine, Chilly-Mazarin, France; 16 Sanofi Research & Development on behalf of Excelya, Montpellier, France; 17 Sanofi, Cambridge, MA USA; 18 Sanofi Oncology, Vitry-sur-Seine, France; 19 University of Nantes, Nantes, France

**Keywords:** Myeloma, Myeloma, Combination drug therapy

## Abstract

Patients with newly diagnosed multiple myeloma (NDMM) ineligible for autologous stem cell transplantation (ASCT) have lower survival rates and may benefit from frontline regimens that include novel agents. This Phase 1b study (NCT02513186) evaluated preliminary efficacy, safety, and pharmacokinetics (PK) of isatuximab, an anti-CD38 monoclonal antibody, combined with bortezomib-lenalidomide-dexamethasone (Isa-VRd) in patients with NDMM ineligible for/with no intent for immediate ASCT. Overall, 73 patients received four 6-week induction cycles of Isa-VRd, then maintenance with Isa-Rd in 4-week cycles. In the efficacy population (*n* = 71), the overall response rate was 98.6%, with 56.3% achieving a complete response or better (sCR/CR), and 36/71 (50.7%) patients reaching minimal residual disease negativity (10^−5^ sensitivity). Grade ≥3 treatment-emergent adverse events (TEAEs) occurred in 79.5% (58/73) of patients but TEAEs leading to permanent study treatment discontinuation were reported in 14 (19.2%) patients. Isatuximab PK parameters were within the previously reported range, suggesting that VRd does not alter the PK of isatuximab. These data support additional studies of isatuximab in NDMM, such as the Phase 3 IMROZ study (Isa-VRd vs VRd).

## Introduction

Despite treatment advances contributing to improved outcomes in patients with multiple myeloma (MM), those not eligible for autologous stem cell transplantation (ASCT) have lower survival rates partially due to receiving less-intensive therapy based on advanced age and/or comorbidities [1, 2]. Achieving minimal residual disease-negative (MRD-) status could represent an early marker of good prognosis in the newly diagnosed MM (NDMM) setting, as studies have shown that it is associated with prolonged progression-free survival (PFS) and overall survival (OS) [3–5].

Molecular and immunophenotypic techniques can be used to monitor MRD [6, 7]. Advances leading to next-generation flow cytometry (NGF) and next-generation sequencing (NGS) resulted in MRD detection at sensitivity thresholds of ≥10^−5^, greatly improving disease cell detection after therapeutic intervention. Accordingly, the International Myeloma Working Group (IMWG) reported new response assessment guidelines [8].

Bortezomib-lenalidomide-dexamethasone (VRd) has become a standard-of-care regimen in patients with NDMM ineligible for/with no intent for immediate ASCT based on the Phase 3 SWOG S0777 study, in which VRd administered until disease progression resulted in significantly longer OS than with lenalidomide-dexamethasone (Rd) alone [9]. Patients ineligible for ASCT may also benefit from backbone regimens plus novel agents (eg, monoclonal antibodies) in the frontline setting, resulting in deep and durable responses [1, 10]. In this regard, incorporation of the anti-CD38 monoclonal antibody daratumumab in the frontline setting with Rd led to a 47% decreased risk of disease progression or death among patients with NDMM ineligible for ASCT [11]. Similarly, adding daratumumab to bortezomib-melphalan-prednisone (VMP) led to a 58% decreased risk of disease progression or death in the same population [12]. Based on these findings, daratumumab combinations were included as standard-of-care regimens in this population.

Adding anti-CD38 monoclonal antibodies (eg, isatuximab) to the VRd standard-of-care regimen may provide additional options for patients. Isatuximab, an IgG1 monoclonal antibody that targets a specific epitope of CD38, has multiple tumor cell–killing mechanisms of action [13–15]. Based on the Phase 3 ICARIA-MM and IKEMA studies, isatuximab is approved in several countries with pomalidomide-dexamethasone for patients with relapsed/refractory MM (RRMM, after ≥2 therapies) or with carfilzomib-dexamethasone for patients with RRMM (United States: 1–3 prior therapies; Japan: 1 prior therapy) or relapsed MM (European Union: ≥1 prior therapy) [16–18].

This study was designed to evaluate the preliminary efficacy, safety, and pharmacokinetics of the quadruplet regimen of isatuximab combined with VRd (Isa-VRd) in transplant-ineligible patients with NDMM. In the Isa-VRd Part B cohort, patients with no intent for immediate transplant were also included.

## Methods

### Study design

**Fig. 1:**
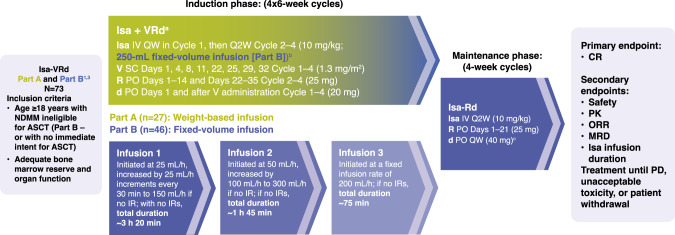
Study design. ^a^Pre-medications included diphenhydramine 25–50 mg IV (or equivalent), dexamethasone 20 mg IV/PO, H2 antagonists, acetaminophen 650–1000 mg PO, montelukast 10 mg PO (or equivalent). The use of montelukast was strongly recommended in Cycle 1, optional from Cycle 2 onward. ^b^Isa 10 mg/kg diluted and administered IV from a fixed-volume infusion bag containing 250 mL of 0.9% sodium chloride solution. ^c^20 mg/day in patients >75 years old. ASCT autologous stem cell transplant, d dexamethasone, h hour, IR infusion reaction, Isa isatuximab, IV intravenously, min minute, MRD minimal residual disease, NDMM newly diagnosed multiple myeloma, ORR overall response rate, PD progressive disease, PK pharmacokinetics, PO orally, QW once weekly, Q2W every other week, R lenalidomide, SC subcutaneous, V bortezomib. At the time of protocol amendment 08 (21 December 2018) implementation, a new cohort (Part B) was included and a new fixed-volume infusion method was tested, including all patients from Part A who had already received Isa using weight-based infusion with the earliest switch at Cycle 19 (Maintenance C15). With the implementation of protocol amendment 11 in October 2021, the schedule of Isa administration in the maintenance phase for both cohorts was changed from every 2 weeks to every 4 weeks (on day 1 of each cycle). This change allowed for alignment with several Isa Phase 3 studies where this switch was implemented in the frontline setting. In trials without the transplant procedure included, this switch is done after at least 12 months of treatment. At the time of this switch, all ongoing 43 (58.9%) patients had received triplet maintenance with Isa every 2 weeks in combination with lenalidomide and dexamethasone for at least 18 months (at least 24 months of treatment in total, as the induction phase duration is 6 months). This study also included a cohort of patients treated with Isa combined with bortezomib, cyclophosphamide, and dexamethasone (Isa-VCd), but safety and efficacy of the Isa-VCd cohort will be published separately.

This open-label, multicenter, Phase 1b study enrolled adults with measurable NDMM ineligible for (Part A and B) or without intent for (Part B) immediate transplantation. Patients with ultra-high–risk smoldering MM fulfilling IMWG criteria were also eligible. Patients were excluded if they had an Eastern Cooperative Oncology Group performance status >2, inadequate liver or renal function, major surgery or radiation therapy within 14 days before study treatment administration, or prior systemic curative treatment for MM. The recruitment periods were May 4, 2017 – April 20, 2018 (Part A) and March 29, 2019 – January 27, 2020 (Part B). The study was conducted in accordance with consensus ethics principles derived from international ethics guidelines, including the Declaration of Helsinki, the International Council for Harmonisation guidelines for Good Clinical Practice, and all applicable laws, rules, and regulations. Written informed consent was obtained prior to conducting any study-related procedures. The study design is shown in Fig. 1. The primary endpoint was complete response (CR) rate. Secondary endpoints consisted of safety; infusion duration of isatuximab; efficacy measured by overall response rate (ORR), duration of response (DOR), and PFS; and the rate of MRD- in patients with CR or very good partial response (VGPR). The efficacy-evaluable population included patients who had completed Cycle 1 and received >2 (weekly [QW]/every 2 weeks [Q2W] schedule) administrations of isatuximab with investigator assessment of response at Cycle 1 or end of treatment with stable disease or better, unless disease progression was diagnosed. Safety analyses were performed on the all-treated population.

### Treatments

Isa-VRd was administered for four 6-week cycles during the induction phase, followed by Isa-Rd (maintenance) until disease progression, unacceptable toxicity, or patient withdrawal. Based on previous findings in relapsed/refractory MM [19], it was considered appropriate to switch to monthly isatuximab administration in the maintenance phase, with all patients receiving at least 2 years of treatment at the time of the switch. In Part A, a weight-based volume infusion of isatuximab 10 mg/kg was used based on the most recent patient weight available on the day of infusion preparation. In the absence of infusion reactions (IRs), durations of the first and subsequent infusions were expected to be ~3 h 20 min and ~2 h 54 min. In Part B, isatuximab 10 mg/kg was diluted and administered intravenously from a fixed-volume infusion bag containing 250 mL of 0.9% sodium chloride solution. In the absence of IRs, durations of the first, second, and third infusions were expected to be ~3 h 20 min, ~1 h 45 min, and ~1 h 15 min, respectively. At the time of analysis, all ongoing patients from Part A had switched to the fixed-volume infusion method.

### Assessments

Disease assessments were performed by investigators every cycle based on local laboratory for serum, urine M-protein, and free light chain (FLC) parameters using IMWG criteria [8].

For patients with suspected isatuximab interference on serum immunofixation (IFE), the Sebia HYDRASHIFT 2/4 Isatuximab IFE test was used by the central laboratory to specifically measure endogenous M-protein. The adjusted response defined the final CR rate per IMWG criteria.

High-risk cytogenetic status was determined based on the presence of del17p, t(4;14), t(14;16), and 1q21+ by both local and central fluorescence in situ hybridization assessments.

MRD was assessed by NGF and NGS at 10^−4^, 10^−^^5^, and 10^−^^6^ by central laboratory. Samples were collected at the time of confirmed VGPR or better, 3 months later, and an additional 3 months later in case of MRD positivity. With the implementation of amendment 10 (December 2020; of 11 total amendments), after initial response of VGPR or better, bone marrow aspirate collection at 1, 2, and 3 years after the first dose were added. Patients with ≥2 sequential MRD- samples were evaluable for sustained MRD-.

Adverse events (AEs) were graded according to the National Cancer Institute Common Terminology Criteria for Adverse Events v4.03.

### Pharmacokinetics

Blood samples were collected at predetermined time points. Isatuximab plasma concentrations were determined with a validated ELISA method (lower limit of quantification, 0.500 ng/mL). Lenalidomide and bortezomib plasma concentrations were determined using validated LC-MS/MS methods (lower limits of quantification, 1.00 ng/mL and 0.500 ng/mL, respectively). PK parameters were calculated based on a non-compartmental analysis (NCA) from the isatuximab, bortezomib, and lenalidomide plasma concentrations obtained after the first and repeated administrations.

### Immunophenotyping

Blood samples were taken at Day 1 of Cycles 1 and 3, and at the end of treatment to explore microenvironment changes. Immune cell populations (ie, CD3, CD4, and CD8 T cell, and NK-cell subsets) were characterized by multiparametric flow cytometry analysis for cell surface marker expression.

### Statistical analysis

For Part A, Simon’s two-stage optimal design was used to determine the sample size. The null hypothesis that the true confirmed CR response rate is 16% was tested against a one-sided alternative. In the first stage, 13 patients were treated. If there were ≤2 confirmed CR response in these 13 patients, the study was to be stopped. Otherwise, 13 additional patients would be treated for a total of 26. The null hypothesis was to be rejected if ≥7 confirmed CR responses were observed in 26 patients. This design yields a Type I error rate of 0.10 and power of 0.80 when the true response rate is 35%.

For Part B, a single-stage, fixed design was used. A total of 44 patients was calculated to provide ≥90% power to reject the null hypothesis that the true response rate is ≤16% when the true response rate is ≥35%, based on a one-sided binomial test with a significance level of 0.05. The null hypothesis was to be rejected if the observed CR rate is ≥25% (11 patients with CR).

Unless otherwise specified, safety analyses were descriptive and performed using the all-treated population. Efficacy endpoints were analyzed using the efficacy population. DOR, PFS, and OS were analyzed using the Kaplan-Meier method.

See Supplementary Materials for additional methods details.

## Results

### Patient baseline characteristics

The median age of all patients was 71.0 (range, 49–87) years, including 15 (20.5%) patients ≥75 years (Table 1). Most patients (91.8%) presented with International Staging System stage I or II, 11 (20.4%) had high cytogenetic risk according to IMWG criteria, and 25 (30.4%) patients presented with 1q21+ abnormalities. Among the 11 patients with high-risk cytogenetics, 5 also had 1q21+ abnormalities.

**Table 1:** Patient demographics and baseline characteristics.

	Isa-VRd^a^		
	Part A	Part B	All
	(*n* = 27)	(*n* = 46)	(*n* = 73)
Age, years			
Median (range)	71.0 (63–77)	70.0 (49–87)	71.0 (49–87)
Age group, *n* (%)			
<65 years	3 (11.1)	8 (17.4)	11 (15.1)
≥65–<75 years	17 (63.0)	30 (65.2)	47 (64.4)
≥75 years	7 (25.9)	8 (17.4)^b^	15 (20.5)
Male, *n* (%)	9 (33.3)	22 (47.8)	31 (42.5)
ECOG PS, *n* (%)			
0	16 (59.3)	23 (50.0)	39 (53.4)
1	9 (33.3)	22 (47.8)	31 (42.5)
2	2 (7.4)	1 (2.2)	3 (4.1)
ISS at study entry, *n* (%)			
Stage I	8 (29.6)	22 (47.8)	30 (41.1)
Stage II	17 (63.0)	20 (43.5)	37 (50.7)
Stage III	2 (7.4)	4 (8.7)	6 (8.2)
MM subtype, *n* (%)			
IgA	5 (18.5)	8 (17.4)	13 (17.8)
IgD	0	1 (2.2)	1 (1.4)
IgG	21 (77.8)	28 (60.9)	49 (67.1)
Kappa light chain only	1 (3.7)	6 (13.0)	7 (9.6)
Lambda light chain only	0	3 (6.5)	3 (4.1)
Measurable paraprotein at baseline^c^, *n* (%)			
Serum M-protein	18 (66.7)	28 (60.9)	46 (63.0)
Urine M-protein	1 (3.7)	4 (8.7)	5 (6.8)
Both serum and urine M-protein	5 (18.5)	8 (17.4)	13 (17.8)
Kappa light chain	1 (3.7)	4 (8.7)	5 (6.8)
Lambda light chain	1 (3.7)	2 (4.3)	3 (4.1)
Cytogenetic risk at study entry, *n* (%)	*n* = 23	*n* = 31	*n* = 54
High^d^	3 (13.0)	8 (25.8)	11 (20.4)
Standard	20 (87.0)	23 (74.2)	43 (79.6)
Molecular subtypes, *n* (%) present			
17p deletion (TP53)	1 (3.7)	4 (8.7)	5 (6.8)
t(4;14)	2 (7.4)	4 (8.7)	6 (8.2)
t(14;16)	0	0	0
1q21+	9 (33.3)	16 (34.8)	25 (34.2)
Median bone marrow plasma cells at study entry, % (range)	17.50 (2.5–90.0)	30.0 (4.0–92.0)	24.5 (2.5–92.0)
Soft tissue plasmacytoma at baseline, *n* (%)	0	2 (4.3)	2 (2.7)
Creatinine clearance, *n* (%)			
<60 mL/min/1.73 m^2^	6 (22.2)	3 (6.5)	9 (12.3)
≥30 to <60 mL/min/1.73 m^2^	6 (22.2)	3 (6.5)	9 (12.3)
<50 mL/min/1.73 m^2^	1 (3.7)	2 (4.3)	3 (4.1)
≥60 mL/min/1.73 m^2^	21 (77.8)	42 (91.3)	63 (86.3)
Missing	0	1 (2.2)	1 (1.4)

*d* dexamethasone, *ECOG PS* Eastern Cooperative Oncology Group performance status, *Ig* immunoglobulin, *Isa* isatuximab, *ISS* International Staging System, *R* lenalidomide, *V* bortezomib.

^a^From May 4, 2017, to April 20, 2018, 27 patients were enrolled in Part A (data cutoff April 5, 2021). From March 29, 2019, to January 27, 2020, 46 patients were enrolled in Part B (data cutoff January 28, 2022).

^b^Of these patients, 4 were >80 years old.

^c^Measurable paraprotein at baseline based on C1D1 value.

^d^High-risk status was determined as having del17p or t(4;14) or t(14;16). Data were based on local FISH assessment when central data were not available for del17p or t(4;14) or t(14;16) and 1q21+ abnormalities. Five patients had combined cytogenetic abnormalities.

### Patient disposition

Of the 27 patients treated in Part A, 13 discontinued treatment due to AEs (*n* = 7; 25.9%), disease progression (*n* = 3; 11.1%), and withdrawal by subject (*n* = 3; 11.1%) (Table S1). Of the 46 patients treated in Part B, 18 discontinued treatments due to AEs (*n* = 8; 17.4%), progressive disease (*n* = 4; 8.7%), poor compliance to protocol (*n* = 1; 2.2%), withdrawal by subject (*n* = 2; 4.3%), and immediate stem cell transplant (*n* = 3; 6.5%). AEs leading to definitive treatment discontinuation are listed in Table S2.

### Infusion duration

In Part A, median duration for the first infusion was 3 h 44 min for patients receiving the weight-based infusion and 3 h 25 min for patients who switched to the fixed-volume infusion method. The median duration decreased to 2 h 49 min and 1 h 48 min for the second infusion, 2 h 45 min and 1 h 18 min for the third infusion, and 2 h 35 min and 1 h 18 min for subsequent infusions.

In Part B, with the fixed-volume infusion method, the median duration of infusion decreased from 3 h 41 min for the first infusion to 1 h 55 min for the second infusion, 1 h 17 min for the third infusion, and 1 h 20 min for subsequent infusions.

### Stem cell mobilization (Part B)

In Part B, 13 (28.3%) patients were eligible but without immediate intent for ASCT and 7 (53.8%) proceeded to stem cell mobilization. Overall, 4 (30.8%) patients proceeded with ASCT: 3 discontinued study treatment to have immediate ASCT per protocol and 1 decided to discontinue study treatment (due to heavy study procedure) and had ASCT during follow-up. Additional details are in the Supplementary Materials.

### Best overall response (BOR)

**Fig. 2:**
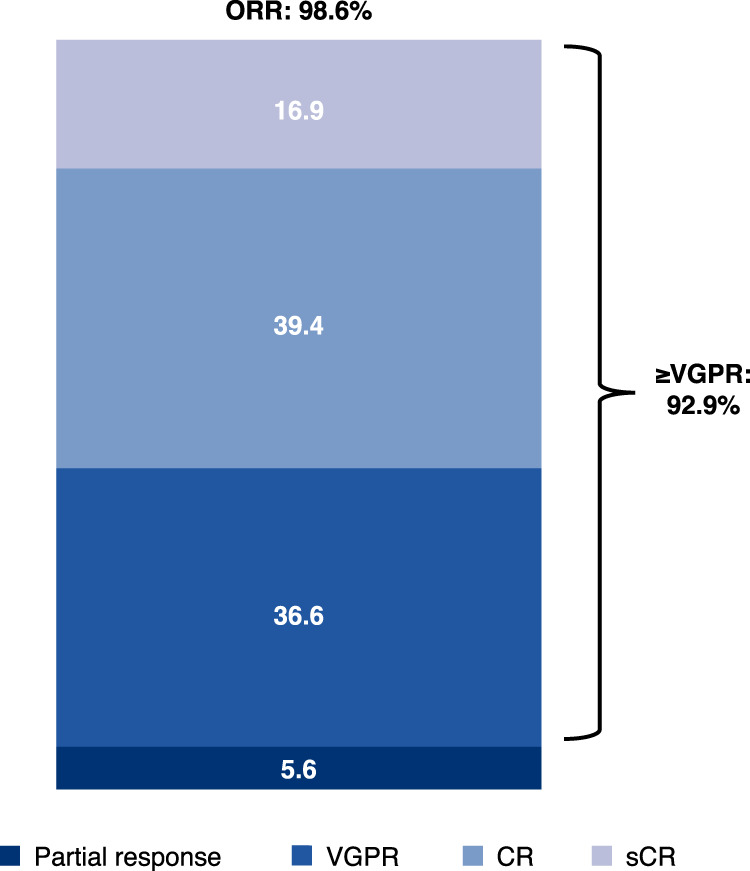
Best overall response in the efficacy population (*n* = 71)^a^. ^a^Data adjusted by incorporating results from 8 (Part A) or 21 (Part B) patients whose samples underwent HYDRASHIFT 2/4 isatuximab IFE testing, an immunofixation test assessing serum M-protein without isatuximab interference. The HYDRASHIFT 2/4 isatuximab IFE assay was launched by Sebia in Europe in February 2021 and approved by FDA in November 2021. CR complete response, IFE immunofixation electrophoresis, sCR stringent complete response, VGPR very good partial response.

For the 71 patients in Parts A and B comprising the efficacy population, the ORR was 98.6% (*n* = 70/71) and the CR or better rate adjusted by the HYDRASHIFT 2/4 isatuximab IFE test was 56.3% (*n* = 40/71) (Fig. 2; Supplementary Materials).

Similar ORRs were seen among patient subgroups, including those with high-risk cytogenetics, older patients, and those with renal impairment (Table S3). Notably, among the 3 patients with renal impairment at baseline (creatinine clearance < 50 mL/min), 2 achieved renal CR (median glomerular filtration rate increased to >60 mL/min/1.73 m^2^) during treatment.

### Minimal residual disease

**Fig. 3:**
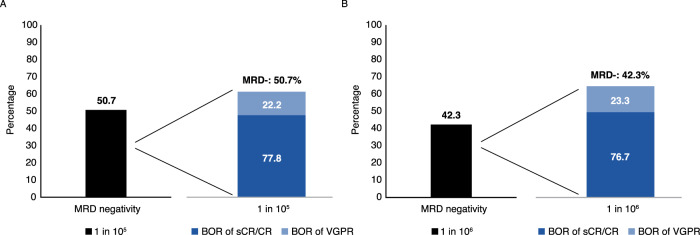
MRD- by BOR in the efficacy population (*n* = 71)^a^. **A** At a sensitivity level of 10^−5^. **B** At a sensitivity level of 10^−6^. ^a^MRD was determined by NGF and NGS methods, and MRD- rate was determined by combining both methods in the case of at least 1 method yielding negative results and the other method showing no positive result at the same time. BOR data adjusted by incorporating results from 8 (Part A) or 21 (Part B) patients whose samples underwent testing with HYDRASHIFT 2/4 isatuximab IFE test, an immunofixation test assessing serum M-protein without isatuximab interference. The HYDRASHIFT 2/4 isatuximab assay was launched by Sebia in Europe in February 2021 and approved by FDA in November 2021. BOR best overall response, CR complete response, MRD- minimal residual disease negativity, NGF next-generation flow cytometry, NGS next-generation sequencing, sCR stringent complete response, VGPR very good partial response.

MRD status was assessed in patients with VGPR or better as BOR. In the efficacy-evaluable population, 36/71 (50.7%) patients achieved MRD- (sensitivity 10^−5^) (Fig. 3A). Of the efficacy-evaluable patients, 28/71 (39.4%) had sCR/CR as BOR, which represents 77.8% (28/36) of patients with MRD- (sensitivity 10^−5^) and sCR/CR. At a sensitivity of 10^−6^, 42.3% achieved MRD- in the efficacy-evaluable population, with 76.7% of patients achieving sCR/CR and MRD- (Fig. 3B).

Among the whole response-evaluable population, similar proportions of patients in Parts A and B achieved MRD- (sensitivity 10^−5^; 46.2% [12/26] and 53.3% [24/45], respectively). Of those achieving MRD-, more patients in Part B vs Part A had a BOR of sCR/CR (83.3% [20/24] vs 66.7% [8/12]).

Among 36 patients with MRD- (sensitivity 10^−5^) based on both NGS and NGF methods, 22 presented with MRD- samples at 2 consecutive time points and were thus evaluable for sustained MRD-. Of these, 5 (22.7%) patients sustained MRD- for 6 months, and 15 (63.6%) sustained MRD- for 1 year (sustained MRD- for 2 years was not yet evaluable at database lock).

### Progression-free survival

**Fig. 4:**
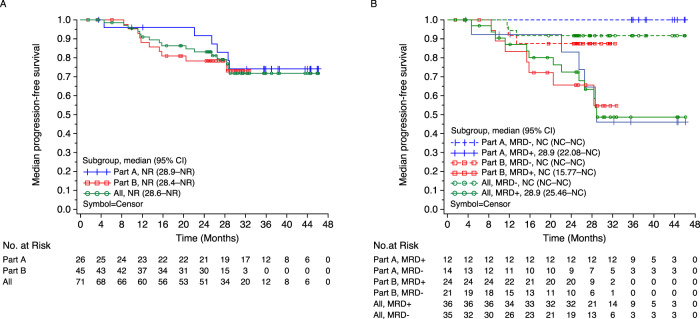
Median PFS at 10^−5^. **A** In the efficacy population. **B** by MRD status^a^. ^a^MRD was determined by NGF and NGS methods, and MRD negativity rate was determined by combining both methods in the case of at least 1 method yielding negative results and the other method showing no positive result at the same time. CI confidence interval, d dexamethasone, Isa isatuximab, MRD minimal residual disease, NC not calculable, PFS progression-free survival, R lenalidomide, V bortezomib.

PFS events occurred in 6/26 patients in Part A (median follow-up, 35.8 months; range, 3.5–46.1) and in 10/45 patients in Part B (median follow-up, 25.3 months; range, 1.5–32.8). Median PFS for the entire cohort was not reached (NR; interquartile range [IQR]: 28.6 months to NR) (Fig. 4A). Examined separately, median PFS estimates were NR (IQR: 28.9 months to NR) for Part A and NR (IQR: 28.4 months to NR) for Part B. The PFS probability at 1 and 2 years for the entire cohort was 91.0% (95% CI: 81.0–95.9) and 83.1% (95% CI: 71.5–90.3), respectively (additional probabilities in Table S4). The estimated PFS rate was numerically higher in patients with MRD-, and an early separation between patients with MRD- and MRD positivity was observed in the KM curves (Fig. 4B).

### Overall survival

**Fig. 5:**
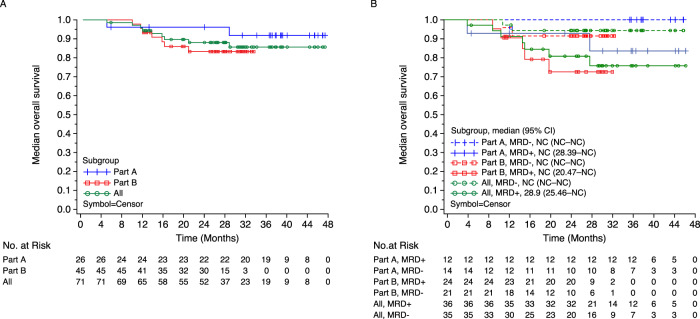
Median OS at 10^−5^. **A** In the efficacy population. **B** by MRD status^a^. ^a^MRD was determined by NGF and NGS methods, and MRD- rate was determined by combining both methods in the case of at least 1 method yielding negative results and the other method showing no positive result at the same time. d dexamethasone, Isa isatuximab, MRD- minimal residual disease negativity, NC not calculable, OS overall survival, R lenalidomide, V bortezomib.

OS events (any cause) occurred in 2/26 patients in Part A (median follow-up, 37.9 months; range, 4.6–47.0) and in 7/45 patients in Part B (median follow-up, 26.2 months; range, 9.5–33.1). Median OS estimates for Parts A and B separately and for the entire cohort were NR (range, NR–NR) (Fig. 5A). The estimated OS rate was numerically higher in patients with MRD- than patients with MRD positivity, and an early separation between patients with MRD- and MRD positivity was observed in the KM curves (Fig. 5B).

### Safety

The median duration of exposure to all drugs was 36.7 (range, 0.2–47.6) months in Part A and 26.5 (range, 0.9–33.2) months in Part B. Median relative dose intensities for each drug are shown in Table 2.

**Table 2:** Median relative dose intensity and duration of exposure of each combination drug.

	Part A	Part B
	(*n* = 27)	(*n* = 46)
Median relative dose intensity, % (range)		
Isatuximab	92.17	89.92^a^
	(23.2–100.3)	(75.0–100.8)
Bortezomib	95.57	89.36
	(67.6–235.8)	(28.6–118.8)
Lenalidomide	72.77	92.25
	(38.1–105.3)	(8.33–123.52)
Dexamethasone	98.31	75.24
	(40.1–247.1)	(23.9–185.2)
Median duration of exposure, weeks (range)		
Isatuximab	159	114.93
	(1.0–206.1)	(3.9–144.3)
Bortezomib	22.43	23.36
	(2.9–28.3)	(3.3–55.7)
Lenalidomide	105.71	109.36
	(15.1–203.3)	(6.0–145.0)
Dexamethasone	123	108.43
	(0.1–201.4)	(3.0–142.4)

^a^The relative dose intensity for isatuximab was lower than expected due to the schedule of isatuximab administration during the maintenance phase being changed from every 2 weeks to every 4 weeks after protocol amendment 11. The change was not incorporated in the calculation and Day 15 was considered as dose omission.

Any-grade TEAEs were reported in 73/73 (100%) patients in the safety population (79.5% Grade ≥3), and 14 (19.2%) patients experienced TEAEs (8/14, Grade ≥3) leading to permanent study treatment discontinuation during a follow-up period of over 2 years (Table 3). TEAEs leading to dose reductions were reported in 25 (92.6%) patients in Part A and 45 (97.8%) patients in Part B. See Supplementary Materials for additional TEAE data for Parts A and B.

**Table 3:** Overview of TEAEs.

*n* (%)	All
	(*n* = 73)
Any TEAE	73 (100)
Grade ≥3 TEAEs	58 (79.5)
Treatment-emergent SAEs	39 (53.4)
Drug-related treatment-emergent SAEs	22 (30.1)
TEAEs leading to death^a^	7 (9.6)
TEAEs leading to permanent discontinuation of study treatment	14 (19.2)
TEAEs leading to premature study drug discontinuation	24 (32.9)
Bortezomib	13 (17.8)
Lenalidomide	11 (15.1)
Dexamethasone	4 (5.5)

^a^TEAEs leading to death included listeremia (*n* = 1), progressive disease (*n* = 1), metastatic breast cancer (*n* = 1), metastatic melanoma (*n* = 1), COVID-19 (*n* = 2), diverticulitis (*n* = 1)

SAE, serious adverse event; TEAE, treatment-emergent adverse event.

In the overall safety population, there were 7 (9.6%) TEAEs leading to death, including COVID-19 (*n* = 2), and listeremia, progressive disease, metastatic breast cancer, metastatic melanoma, and diverticulitis (*n* = 1 each). The most common TEAEs occurring in at least 20% of patients included constipation (68.5%), diarrhea (64.4%), asthenia (63.0%), peripheral sensory neuropathy (61.6%), peripheral edema (46.6%), and IRs (41.1%) (Table S5). The most frequently reported TEAEs of any severity, and Grade ≥3 TEAEs reported in >1 patient are listed in Tables S6 and S7.

TEAEs of infections of any grade occurred in 60 (82.2%) patients and Grade ≥3 events in 17 (23.3%) patients, including bronchitis (*n* = 3; 4.1%), and cellulitis, COVID-19, and pneumonia/pneumonia haemophilus (*n* = 2; 2.7% each).

Overall, 15 (55.6%) patients in Part A and 29 (63.0%) patients in Part B received antibiotic prophylaxis (mainly sulfamethoxazole-trimethroprim, amoxicillin, and levofloxacin).

Overall, 73.3% of IRs occurred during the first infusion in both Part A and Part B. IRs occurred in 17 (63.0%) patients in Part A and in 13 (28.3%) patients in Part B, with most being Grade 2. A single Grade 3 IR occurred in Part A resulting in discontinuation, whereas no Grade ≥3 IRs were reported in Part B. The difference in IRs between Parts A and B can be partially explained by montelukast use. In Part A, 2 (7.4%) patients received montelukast as premedication; among them, 1 (50.0%) had an IR. In Part B, 31 (67.4%) patients received montelukast as premedication; among them, 7 (22.6%) had an IR.

In Part A, hematologic laboratory abnormalities of all grades occurred during the treatment period in all patients except for neutropenia, which occurred in 77.8% of patients. Grade 3–4 abnormalities were reported for lymphopenia (*n* = 22; 81.5%), neutropenia (*n* = 14; 51.8%), thrombocytopenia (*n* = 10; 37.0%), leukopenia (*n* = 9; 33.3%), and anemia (*n* = 2; 7.4%).

In Part B, hematologic laboratory abnormalities of all grades occurred during the treatment period for anemia (46/46; 100%), lymphopenia and leukopenia (45/46; 97.8% each), neutropenia (41/46; 89.1%), and thrombocytopenia (40/46; 87.0%). Grade 3-4 abnormalities were reported for lymphopenia (*n* = 35; 76.1%), neutropenia (*n* = 20; 43.4%), thrombocytopenia (*n* = 16; 34.7%), leukopenia (*n* = 16; 34.7%), and anemia (*n* = 5; 10.9%).

### Pharmacokinetics

Based on results from the PK population for NCA on Cycle 1, when given in combination with VRd, isatuximab PK parameters (area under the plasma concentration versus time curve from 0 to 1 week [AUC_0-1week_]) were within the range of those previously reported [20, 21], suggesting that VRd does not alter the PK of isatuximab (Table S8). Similarly, both PK parameters of bortezomib and lenalidomide were consistent with those previously reported in the literature [22–25].

### Immunophenotyping

Flow cytometry experiments were conducted to explore changes in the immune microenvironment upon treatment. Data from Parts A and B were obtained from peripheral blood collected at Day 1 of Cycle 1 (27 and 43 patients, respectively), Day 1 of Cycle 3 (20 and 38 patients), and end of treatment (8 and 9 patients) for the all-treated population.

For the all-treated population, at Day 1 of Cycle 3 compared with baseline, the percentages of CD19 + B cells (2.33% to 0.66%), CD4 + T cells (15.56% to 11.27%), CD3 + T cells (23.56% to 19.00%), NK cells (CD56^bright^ CD16^low^, 2.01% to 0.26%; CD56^dim^ CD16^bright^, 1.32% to 0.26%), and T-regulatory cells (2.06% to 0.74%) were decreased in the peripheral blood. The percentage of CD4 + T cells, CD3 + T cells, and T-regulatory cells came back to baseline levels (or above) at the end of treatment. At the end of treatment compared with baseline, the percentages of CD19 + B cells (2.33% to 0.97%) and NK cells (CD56^bright^ CD16^low^, 2.01% to 0.45%; CD56^dim^ CD16^bright^, 1.32% to 0.43%) were still decreased. Compared with baseline, CD8 + T cell percentage was increased in the peripheral blood at the end of treatment (6.62% to 12.47%) (Fig. S1).

As all patients except 1 responded to the treatment, no correlation with parameters of clinical response was performed.

## Discussion

This Phase 1b study was designed to investigate Isa-VRd for the first time in adult patients with NDMM who were ineligible/had no intent for immediate transplantation. Treatment with isatuximab plus triplet bortezomib-lenalidomide-dexamethasone resulted in deep and durable responses, with an MRD- rate of 51% at a sensitivity of 10^−5^ in the whole population. The ORR was 98.6% (70/71) and the CR or better rate adjusted by the HYDRASHIFT 2/4 isatuximab IFE test was 56.3% (40/71). The estimated PFS rate was numerically higher in patients with MRD- than in patients with MRD positivity, and an early separation between patients with MRD- and MRD positivity was observed in the KM curves.

Median PFS in the overall population of the current study was not reached (NR; interquartile range [IQR], 28.6 months to NR). The PFS probability at 1 and 2 years for the entire cohort was 91.0% (95% CI: 81.0–95.9) and 83.1% (95% CI: 71.5–90.3), respectively. Examined separately, median PFS estimates were NR (IQR: 28.9 months to NR) for Part A (median follow-up, 35.8 months) and NR (IQR: 28.4 months to NR) for Part B (median follow-up, 25.3 months). Results from the Phase 3 SWOG S0777 study demonstrated significantly improved PFS with VRd vs Rd (43 vs 30 months; stratified HR 0.712 [95% CI: 0.56–0.906]) [9]. The 1- and 2-year PFS estimates from the KM curves for those receiving VRd were 90% and 70%, respectively, reflecting similar 1-year and slightly better 2-year PFS estimates for the current study investigating the Isa-VRd quadruplet regimen.

Daratumumab is another anti-CD38 monoclonal antibody used for the treatment of patients with NDMM. In the Phase 3 MAIA study, after a median follow-up of 28.0 months, median PFS was NR in the daratumumab-Rd group vs 31.9 months in the Rd group (HR 0.56; 95% CI, 0.43–0.73) [26]. The 1- and 2-year PFS estimates from the KM curves for those receiving daratumumab were 90% and 80%, respectively, which are similar to the estimates in the current study that had longer follow-up.

In the MAIA and ALCYONE studies, daratumumab plus Rd or bortezomib-melphalan-prednisone (VMP) led to increased MRD- rates, assessed using NGS at a sensitivity of 10^−5^, compared with standard of care (28.8% [D-Rd] vs 9.2% [Rd]; 26.9% [D-VMP] vs 7.0% [VMP]) in patients with transplant-ineligible NDMM [27]. Among these patients, those who achieved MRD- had improved clinical outcomes and deep remission. The current study of Isa-VRd in patients with NDMM ineligible/with no immediate intent for transplant demonstrated MRD- rates of approximately 50.7%, the highest reported so far in this population, and a VGPR or better rate of 92.9%, highlighting the benefit of this quadruplet combination in this patient population to achieve deep remission and improved clinical outcomes. These encouraging data support additional studies of isatuximab quadruplet combinations in the NDMM setting. Ongoing Phase 3 studies include NCT03319667 (IMROZ) and NCT03617731 (GMMG-HD7), with the objective to explore the same quadruplet in both transplant-ineligible and eligible patients, respectively.

In the combined population of Parts A and B, there were no new safety concerns related to isatuximab compared with the results of the ICARIA-MM and IKEMA studies in RRMM [28, 29]. Despite the older median age, study drug exposure was encouraging. The rate of infections is notable (82.2%, all Grades; 23.3%, Grade ≥3) despite the use of antibiotic prophylaxis in over half of the patients. The most frequent infections were respiratory, with upper respiratory tract infection, bronchitis, and nasopharyngitis each being reported in over 25% of patients. However, of these, only 3 cases (4.1%, all bronchitis) were Grade ≥3. Seven infections led to definitive treatment discontinuation (1 pneumonia, 2 COVID-19, 1 listeremia, 2 diverticulitis, and 1 encephalitis). The safety profile, and more specifically, the incidence of severe peripheral neuropathy, infections, or IRs was favorable to this combination compared with existing data of anti-CD38-based triplets or quadruplets in the frontline setting in the same patient population. In the SWOG S0777 study, Grade ≥3 neurological AEs resulting from the use of intravenous bortezomib twice weekly were common, at 33% vs 11% with Rd alone, and contributed to discontinuation of the VRd combination [9]. In the current study, bortezomib was delivered using the current subcutaneous method, which led to a decrease in Grade ≥3 neurological AEs, at 21.9% for Parts A and B combined.

Overall, the incidence and severity of IRs were comparable to those reported with isatuximab in RRMM patients [28, 29]. However, there were fewer IRs of any grade reported in Part B (13 [28.3%] patients), and no Grade ≥3 events, compared with Part A (17 [63%] patients, one Grade ≥3 event) and in previous isatuximab trials [28, 29]. The IR incidence decrease could be partially explained by montelukast use as premedication (67.4% in Part B, 7.4% in Part A). The impact of the COVID-19 pandemic should also be considered in Part A and B, as treatment continued through the data cutoff of April 2021 (Part A). Due to the staggered enrollment periods of Part A and Part B, ending in January 2020, more patients in Part A were receiving maintenance treatment that required less frequent clinic visits, whereas more patients in Part B receiving induction treatment may have experienced pandemic-related challenges based on the closer proximity to the start of the pandemic.

Based on PK results from Parts A and B, when given in combination with VRd, isatuximab PK exposure was within the range of those previously reported, suggesting that VRd does not alter the PK of isatuximab [20, 21]. Similar results to those previously published were also observed for PK parameters of bortezomib and lenalidomide [22–25]. Overall, the exposures of isatuximab, lenalidomide, and bortezomib did not appear to be altered when given in combination, suggesting the lack of drug-drug interactions between these agents. Data investigating the impact of immunogenicity on safety, efficacy, and PK profile are forthcoming.

This study also evaluated isatuximab infusion duration. With the fixed-volume infusion, patients in Part A who switched from the weight-based infusion method and all patients in Part B exhibited a larger decrease in the median infusion time from ~3 h 40 min during the first infusion to ~1 h 20 min after the third infusion compared with patients in Part A with the weight-based infusion method ( ~ 3 h 40 min to ~2 h 35 min), with no increase in IRs reported.

Stem cell collection showed adequate yield of cells/kilogram of body weight, which is required for further successful engraftment.

Descriptive analysis of blood immune cell subpopulations in our study showed a decrease in CD3 and CD4 T cells, NK cells, regulatory T cells, and B cells at Cycle 3, in agreement with previous observations following single-agent treatment with an anti-CD38 antibody [30]. Following combination therapy, our results suggest that Isa-VRd may be associated with T-cell–mediated immune activity and a reduction in T-cell immunosuppressive mechanisms in patients with MM.

In conclusion, this Phase 1b study demonstrated for the first time that a quadruplet regimen of isatuximab, an anti-CD38 monoclonal antibody, plus VRd led to deep responses in patients with NDMM ineligible/with no immediate intent for ASCT, including a 51% MRD- rate. In addition, Isa-VRd exhibited a safety profile consistent with that of each individual drug, suggesting that this quadruplet combination is both feasible and effective in this patient population. The ongoing Phase 3 BENEFIT study (NCT04751877) was designed to investigate isatuximab-based triplet (Isa-Rd) vs quadruplet (Isa-VRd) regimens in patients with NDMM ineligible for ASCT. This study will help determine any added value of the quadruplet vs triplet regimen in this patient population, considering the safety profile of each regimen.

## Supplementary information

Supplementary Material

## Data Availability

Qualified researchers can request access to patient-level data and related study documents including the clinical study report, study protocol with any amendments, blank case report forms, statistical analysis plan, and dataset specifications. Patient-level data will be anonymized, and study documents will be redacted to protect the privacy of trial participants. Further details on Sanofi’s data-sharing criteria, eligible studies, and process for requesting access are at: https://www.vivli.org.
