# Supplementary material for: Efficacy and safety of isatuximab plus bortezomib, lenalidomide, and dexamethasone in patients with newly diagnosed multiple myeloma ineligible/with no immediate intent for autologous stem cell transplantation
Source: Leukemia. 2023 Jun 14;37(7):1521–9. doi: 10.1038/s41375-023-01936-7 (PMC10264885; doi:10.1038/s41375-023-01936-7)
Supplement: Supplementary file 1 — Supplementary Material [file 41375_2023_1936_MOESM1_ESM.pdf]

SUPPLEMENTARY MATERIALS

Methods

Study design

Measurable multiple myeloma (MM) was defined as the presence of at least one of the following: serum M-protein ≥1 g/dL, urine M-protein ≥200 mg/24 h, or serum free light chain (FLC) assay with involved FLC assay ≥10 mg/dL plus an abnormal serum FLC ratio (<0.26 or >1.65).

In Part A, starting infusion rates for isatuximab include the following:

- First infusion: 175 mg/h. In the absence of infusion reactions (IRs) after 1 h, increase by 50 mg/h every 30 min (max: 400 mg/h).
- Subsequent infusions: 175 mg/h. In the absence of IRs after 1 h, increase by 100 mg/h every 30 min (max: 400 mg/h).

In Part B, starting infusion rates for isatuximab include the following:

- First infusion: 25 mL/h. In the absence of IRs after 1 h, increase by 25 mL/h every 30 minutes (max: 150 mL/h). In case of no IRs, total duration of infusion is ~3 h 20 min.
- Second infusion: 50 mL/h. In the absence of IRs after 30 min, increase by 50 mL/h during the next 30 min; then by 100 mL/h during each subsequent 30 min (max: 300 mL/h) until total volume is infused. In case of no IRs, total duration of infusion is ~1 h 45 min.
- Third and subsequent infusions: fixed infusion rate of 200 mL/h. In case of no IRs, total duration of infusion is ~1 h 15 min.

Assessments

When done centrally, the characterization of cytogenetic risk of the myeloma cells was performed by fluorescence in situ hybridization (FISH) testing after immunomagnetic isolation of CD138+ plasma cells from baseline bone marrow aspirate, interphase chromosome preparation, and hybridization with Abbott Molecular FISH probes (Vysis TP53/CEP 17 FISH Probe, Vysis IGH/FGFR3 DF FISH Probe, Vysis IGH/MAF DF FISH Probe) (Des Plaines, IL) and MetaSystems probe (XL CDKN2C/CKS1B FISH probe) (Altlussheim, Germany). An abnormality was considered positive if it was present in at least 10% of analyzed plasma cells.

The safety profile was assessed based on findings of physical examination, laboratory tests, and reports of adverse events (AEs), and was based on the incidence, severity, and cumulative nature of treatment-emergent AEs (TEAEs).

The baseline for a given parameter was defined as the last assessment for the parameter performed before or on the date of the first study treatment.

Results

Patient disposition

Patient disposition is shown in **Table S1**. AEs leading to definitive treatment discontinuation in Parts A and B are shown in **Table S2**.

Table S1. Patient disposition.

	**Isa-VRd**		
	**Part A (*n* = 27)**	**Part B (*n* = 46)**	**All (*n* = 73)**
**Ongoing Treatment**	14 (51.9)	28 (60.9)	42 (57.5)
**Off treatment**	13 (48.1)	18 (39.1)	31 (42.5)
Reason for definitive treatment discontinuation			

Adverse event	7 (25.9)	8 (17.4)	15 (20.5)
Progressive disease	3 (11.1)	4 (8.7)	7 (9.6)
Withdrawal by subject	3 (11.1)	2 (4.3)	5 (6.8)
Poor compliance to protocol	0	1 (2.2)	1 (1.4)
Immediate stem cell transplant	0	3 (6.5)	3 (4.1)

d, dexamethasone; Isa, isatuximab; R, lenalidomide; V, bortezomib.

Table S2. Adverse events leading to definitive treatment discontinuation

	Adverse event	Grade	Relationship to treatment
**Part A**	Encephalitis	≥3	Isatuximab, bortezomib, lenalidomide, dexamethasone
	Listeremia	≥3	Lenalidomide, dexamethasone
	Neutropenia	≥3	No
	Dyspnea	≥3	No
	Pneumonia	1	Isatuximab, lenalidomide
	Fatigue	1	No
	Infusion reaction	≥3 (*n* = 1)	Isatuximab
**Part B**	COVID-19	3 (*n* = 1); 4 (*n* = 1)	No
	Diverticulitis	2	No
	Metastatic malignant melanoma	3	No
	Peripheral sensory neuropathy	2	Lenalidomide
	Cerebral venous sinus thrombosis	2	Lenalidomide
	Toxic skin eruption	1	Lenalidomide
	Hepatocellular injury in the context of metastatic breast cancer	3	No

COVID-19, coronavirus disease 2019.

Stem cell mobilization (Part B)

At the time of mobilization, the best overall response (BOR) was VGPR for 5 (71.4%) patients and PR for 2 (28.6%) patients. In total, 6 (85.7%) patients underwent apheresis (median 8.1 × 10^6 CD34+ cells/kg collected; range, 2–11 × 10^6 CD34+ cells/kg) and 1 patient had stem cell mobilization failure. Agents used for mobilization for patients who underwent apheresis were granulocyte colony-stimulating factor (G-CSF; *n* = 2; 33.3%), or both G-CSF and Plerixafor (*n* = 4; 66.6%). Mobilization by chemotherapy was not allowed. Median time from first dose to apheresis was 20.8 weeks (range, 13.1–42.6 weeks). The reason for patients not proceeding to apheresis was mainly due to medical decisions based on patient age and further response to study treatment.

Before being transplanted, 2 patients had a BOR of PR and 2 patients had an adjusted BOR of VGPR or CR, respectively. Median time from first study treatment to ASCT was 7.0 months (range, 7–17 months). Median time to hematopoietic recovery was 10 days (range, 0–15 days) and 11.0 days (range, 10–12 days) for platelets and absolute neutrophil count, respectively

Best overall response

Of the 26 patients in the efficacy population of Part A, the ORR adjusted using the isatuximab Hydrashift 2/4 IFE test was 100% and the CR or better rate was 50%. The ORR was 97.8% (44/45) in the efficacy population of Part B, with a CR or better rate of 60.0% (27/45).

Similar results were seen in the overall efficacy population among different subgroups of patients (**Table S3**). These response rates were not adjusted using the isatuximab Hydrashift 2/4 IFE test.

Table S3. Response rates among patient subgroups.

Patient subgroup	n	ORR	CR or better rate

High-risk cytogenetics	10	90.0%	60.0%
Age <75 years	57	98.2%	49.1%
Age ≥75 years	14	100%	42.9%
R-ISS Stage I	24	100%	45.8%
R-ISS Stage II	45	100%	48.9%
ECOG PS 0-1	68	98.5%	47.1%
Creatinine clearance ≥60 mL/min/1.73 m^2	61	98.4%	45.9%
Creatinine clearance <60 mL/min/1.73 m^2	9	100%	55.6%

CR, complete response; ECOG PS, Eastern Cooperative Oncology Group performance status; ORR, overall response rate, R-ISS, revised International Staging System.

Progression-free survival

Additional progression-free survival probabilities are shown in **Table S4.**

Table S4. Probabilities of progression-free survival in the efficacy population.

Probability of surviving (95% CI)	Part A (*n* = 26)	Part B (*n* = 45)	All (*n* = 71)
**4 months**	1.000 (1.000 to 1.000)	1.000 (1.000 to 1.000)	1.000 (1.000 to 1.000)
**8 months**	0.960 (0.748 to 0.994)	1.000 (1.000 to 1.000)	0.985 (0.900 to 0.998)
**12 months**	0.960 (0.748 to 0.994)	0.881 (0.737 to 0.949)	0.910 (0.810 to 0.959)
**16 months**	0.960 (0.748 to 0.994)	0.810 (0.655 to 0.900)	0.864 (0.754 to 0.927)
**20 months**	0.960 (0.748 to 0.994)	0.810 (0.655 to 0.900)	0.864 (0.754 to 0.927)
**24 months**	0.916 (0.704 to 0.978)	0.783 (0.625 to 0.881)	0.831 (0.715 to 0.903)

**28 months**	0.829 (0.606 to 0.932)	0.783 (0.625 to 0.881)	0.790 (0.665 to 0.873)
**32 months**	0.742 (0.513 to 0.875)	0.731 (0.542 to 0.852)	0.718 (0.575 to 0.820)
**36 months**	0.742 (0.513 to 0.875)	0.731 (0.542 to 0.852)	0.718 (0.575 to 0.820)
**40 months**	0.742 (0.513 to 0.875)	0.731 (0.542 to 0.852)	0.718 (0.575 to 0.820)
**44 months**	0.742 (0.513 to 0.875)	0.731 (0.542 to 0.852)	0.718 (0.575 to 0.820)
**48 months**	0.742 (0.513 to 0.875)	0.731 (0.542 to 0.852)	0.718 (0.575 to 0.820)

CI, confidence interval.

Safety

TEAEs reported in ≥20% of patients in the all-treated population are shown in **Table S5**.

Table S5. TEAEs reported in ≥20% of patients, all-treated population.

Primary system organ class<br>Preferred term, n (%)	All<br>(*n* = 73)	
	All grades	Grade ≥3
**Infections and infestations**	60 (82.2)	17 (23.3)
Upper respiratory tract infection	19 (26.0)	0
Bronchitis	18 (24.7)	3 (4.1)
Nasopharyngitis	18 (24.7)	0
**Blood and lymphatic system disorders**	23 (31.5)	16 (21.9)
Neutropenia	15 (20.5)	12 (16.4)
**Metabolism and nutrition disorders**	26 (35.6)	3 (4.1)
Decreased appetite	15 (20.5)	0
**Psychiatric disorders**	35 (47.9)	5 (6.8)
Insomnia	19 (26.0)	4 (5.5)
**Nervous system disorders**	67 (91.8)	16 (21.9)
Peripheral sensory neuropathy	45 (61.6)	4 (5.5)

Dizziness	19 (26.0)	0
Headache	17 (23.3)	1 (1.4)
**Eye disorders**	34 (46.6)	5 (6.8)
Cataract	21 (28.8)	3 (4.1)
**Vascular disorders**	37 (50.7)	9 (12.3)
Hypotension	15 (20.5)	1 (1.4)
**Respiratory, thoracic, and mediastinal disorders**	47 (64.4)	10 (13.7)
Dyspnea	20 (27.4)	4 (5.5)
Cough	20 (27.4)	0
**Gastrointestinal disorders**	65 (89.0)	9 (12.3)
Constipation	50 (68.5)	1 (1.4)
Diarrhea	47 (64.4)	8 (11.0)
Nausea	17 (23.3)	0
**Skin and subcutaneous tissue disorders**	40 (54.8)	3 (4.1)
Rash	16 (21.9)	2 (2.7)
**Musculoskeletal and connective tissue disorders**	59 (80.8)	9 (12.3)
Back pain	24 (32.9)	3 (4.1)
Pain in extremity	19 (26.0)	0
Arthralgia	15 (20.5)	0
**Renal and urinary disorders**	15 (20.5)	1 (1.4)
**Injury, poisoning, and procedural complications**	50 (68.5)	3 (4.1)
Infusion-related reaction	30 (41.1)	1 (1.4)
Accidental overdose	15 (20.5)	1 (1.4)
**General disorders and administration site conditions**	66 (90.4)	12 (16.4)
Asthenia	46 (63.0)	7 (9.6)
Peripheral edema	34 (46.6)	2 (2.7)
Fatigue	19 (26.0)	1 (1.4)
Pyrexia	18 (24.7)	0
**Investigations**	19 (26.0)	6 (8.2)

In Part A, TEAEs leading to dose reductions in >10% of patients included peripheral sensory neuropathy (44.4%), cataract and peripheral edema (22.2% each), bronchitis, neutropenia, insomnia, and asthenia (18.5% each), and dyspnea, diarrhea, and tremor (11.1% each).

In Part B, TEAEs leading to dose reductions in >10% of patients included asthenia (32.6%), peripheral sensory neuropathy and diarrhea (30.4%), neutropenia (17.4%), peripheral sensorimotor neuropathy (15.2%), and thrombocytopenia (10.9%).

For Parts A and B, the most frequently reported (>25% of patients) TEAEs of any severity, regardless of causal relationship with study treatment, and Grade ≥3 TEAEs reported in >1 patient are shown in **Table S6** and **Table S7**.

Table S6. Most frequently reported (>25% of patients) TEAEs of any severity, regardless of causal relationship with study treatment, n (%).

TEAE	Part A (*n* = 27)	Part B (*n* = 46)
Peripheral sensory neuropathy	20 (74.1)	25 (54.3)
Diarrhea	18 (66.7)	29 (63.0)
Constipation	18 (66.7)	32 (69.6)
Infusion reaction	17 (63.0)	13 (28.3)
Peripheral edema	16 (59.3)	18 (39.1)
Asthenia	15 (55.6)	31 (67.4)
Cough	12 (44.4)	-
Back pain	11 (40.7)	13 (28.3)
Hypotension	11 (40.7)	-
Fatigue	11 (40.7)	-
Pyrexia	11 (40.7)	-
Upper respiratory tract infection	11 (40.7)	-

Bronchitis	10 (37.0)	-
Dyspnea	10 (37.0)	-
Nasopharyngitis	10 (37.0)	-
Arthralgia	9 (33.3)	-
Dizziness	9 (33.3)	-
Cataract	8 (29.6)	13 (28.3)
Headache	8 (29.6)	-
Accidental overdose	7 (25.9)	-
Musculoskeletal chest pain	7 (25.9)	-
Myalgia	7 (25.9)	-
Insomnia	-	13 (28.3)
Pain in extremity	-	13 (28.3)

TEAE, treatment-emergent adverse event.

Table S7. Grade ≥3 TEAEs reported in >1 patient, n (%).

TEAE	Part A (*n* = 27)	Part B (*n* = 46)
Neutropenia	5 (18.5)	7 (15.2)
Diarrhea	4 (14.8)	4 (8.7)
Peripheral sensory neuropathy	3 (11.1)	2 (4.3)
Asthenia	3 (11.1)	4 (8.7)
Bronchitis	3 (11.1)	-
Dyspnea	3 (11.1)	-
Cataract	3 (11.1)	-
Back pain	2 (7.4)	-
Insomnia	2 (7.4)	-
Hypertension	2 (7.4)	-
Cellulitis	2 (7.4)	-
Thrombocytopenia	-	4 (8.7)
Bone pain	-	3 (6.5)

Peripheral edema	-	2 (4.3)
Deep vein thrombosis	-	2 (4.3)
Insomnia	-	2 (4.3)
COVID-19	-	2 (4.3)
Hepatic cytolysis	-	2 (4.3)
Pulmonary embolism	-	2 (4.3)

COVID-19, coronavirus disease 2019; TEAE, treatment-emergent adverse event.

Pharmacokinetics

A summary of PK parameters is shown in **Table S8**.

Table S8. Summary of PK parameters after the first administration of isatuximab, bortezomib, and lenalidomide in Parts A and B – Cycle 1.

Mean ± SD	Part A	Part B
**Isatuximab (10 mg/kg QW/Q2W)**	*N* = 19	*N* = 32
C_{max} (µg/mL)	171 ± 66.8	179 ± 61.6
Geometric mean	159	169
t_{max}[a] (hr)	4.27	3.95
Range	2.42–24.02	3.33–24.2
$AUC_{0\text{-}1week}$ (µg•hr/mL)	13300 ± 4370	14300 ± 4460[c]
Geometric mean	12600	13700
**Lenalidomide (25 mg)**	*N* = 4	*N* = 24

$AUC_{0\text{-}24h}$ (ng•hr/mL)	2530 ± 533	3200 ± 1370
Geometric mean	2490	2940
CL/F (L/hr)	10.2 ± 2.33	9.05 ± 3.36[d]
Geometric mean	9.99	8.44
**Bortezomib (1.3 mg/m^2)**	*N* = 6	*N* = 21
C_{max} (ng/mL)	18.4 ± 5.45	20.6 ± 11.7
Geometric mean	17.9	17.7
$AUC_{0\text{-}72h}$ (ng•hr/mL)	62.5 ± 29.6[b]	72.1 ± 40.4[e]
Geometric mean	56.7	63.5

[a]Median; [b]N = 5; [c]N = 30; [d]N = 18; [e]N = 15.

$AUC_{0\text{-}1week}$, area under the plasma concentration versus time curve from 0 to 1 week; $AUC_{0\text{-}24h}$, area under the plasma concentration versus time curve from 0 to 24h; CL/F, drug clearance; $AUC_{0\text{-}72h}$, area under the plasma concentration versus time curve from 0 to 72h; C_{max}, maximum plasma concentration observed; QW, weekly; Q2W, every 2 weeks; SD, standard deviation; t_{max}, time to reach C_{max}.

Immunophenotyping

Blood cell immunophenotyping results are shown in **Figure S1**.

Figure S1. Blood cell immunophenotyping results at baseline (*n* = 65 to 70), Day 1 of treatment Cycle 3 (*n* = 47 to 58), and end of treatment (*n* = 16 to 17) expressed as percentage of leukocytes

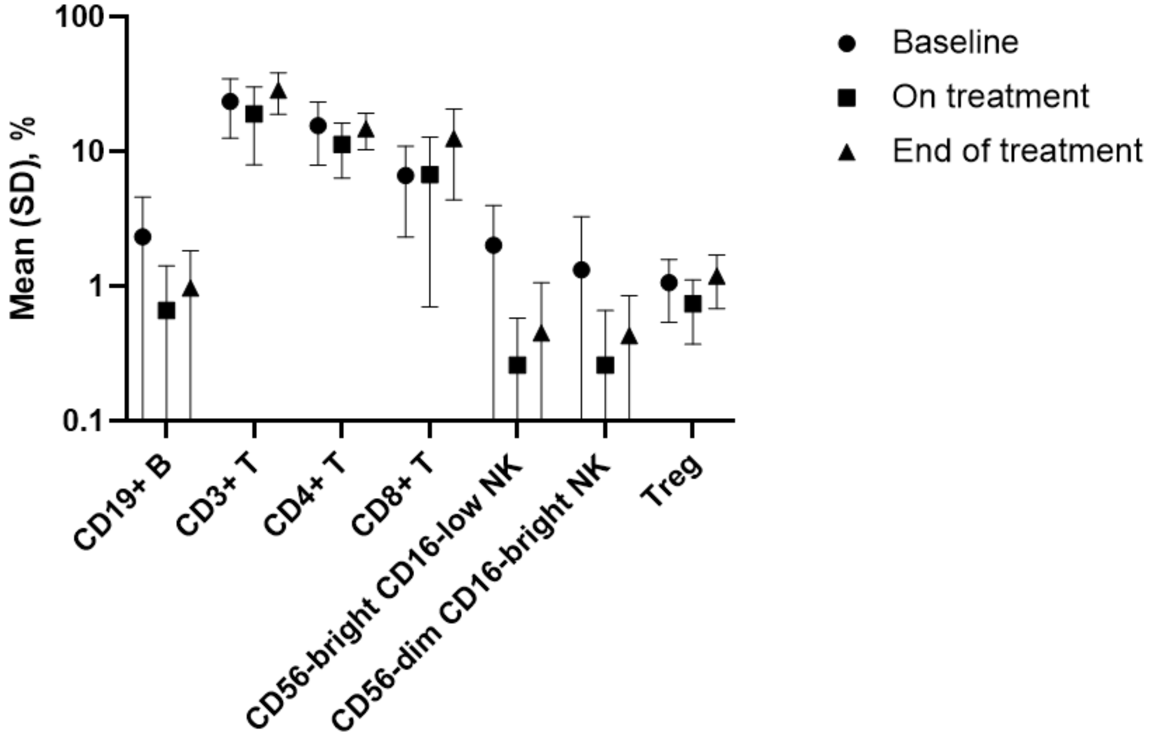

d, dexamethasone; Isa, isatuximab; NK, natural killer; R, lenalidomide; SD, standard deviation; Treg, regulatory T cell; V, bortezomib.
